# Genomic Insights and Antifungal Efficacy of *Xenorhabdus budapestensis* XH-4 in Combating Soybean Root Rot

**DOI:** 10.3390/jof12050332

**Published:** 2026-05-02

**Authors:** Yafei Qu, Kebin Li, Zhimin Wang, Huanhuan Dong, Athanase Hategekimana, Xiaomei Wang, Jiao Yin

**Affiliations:** 1 Department of Plant Pathology, College of Plant Protection, Jilin Agricultural University, No. 2888, Xincheng Street, Changchun 130118, China; qyf18730281819@163.com (Y.Q.); wangzm711@126.com (Z.W.); 15248275498@163.com (H.D.); 2 State Key Laboratory for Biology of Plant Diseases and Insect Pests, Institute of Plant Protection, Chinese Academy of Agricultural Sciences, Yuanmingyuan West Road, Beijing 100193, China; kbli@ippcaas.cn; 3 Crop Protection Program, Rwanda Agriculture and Animal Resources Development Board (RAB), Kigali P.O. Box 5016, Rwanda; athanase.hategekimana@rab.gov.rw

**Keywords:** *Glycine max*, root rot, *Xenorhabdus budapestensis*, biocontrol, *Fusarium oxysporum*

## Abstract

Soybean root rot, primarily caused by *Fusarium oxysporum*, leads to severe root decay and substantial yield losses in *Glycine max*. This study screened ten entomopathogenic nematode-associated symbiotic bacteria for antagonistic activity against *F. oxysporum*. Among them, *Xenorhabdus budapestensis* XH-4 exhibited the strongest in vitro inhibition, suppressing mycelial growth by more than 73%. Antifungal activity was primarily attributed to extracellular metabolites, as both fermentation broth and cell-free culture supernatant were effective, whereas bacterial cell suspensions showed no significant inhibition. In greenhouse experiments, 40% (*v*/*v*) XH-4 reduced the disease index by 75–80%, comparable to the chemical fungicide metalaxyl–hymexazol. Genome mining revealed 20 biosynthetic gene clusters encoding diverse secondary metabolites, including fabclavine, fabclavine pyrrolizixenamide A, and putrebactin/avaroferrin, which likely underpin the strain antifungal efficacy. Additionally, XH-4 enhanced soybean antioxidant capacity and activated the phenylpropanoid pathway, indicating a dual mechanism involving direct antagonism and induced systemic resistance. These findings support the development of XH-4 as an environmentally friendly biofungicide for sustainable management of soybean root rot.

## 1. Introduction

*Glycine max* is a vital food and oilseed crop, but its production is severely threatened by soybean root rot, a widespread soil-borne disease that causes about 20% yield loss and can even result in total crop failure [1,2]. The disease is caused by diverse pathogens such as *Fusarium*, *Phytophthora*, *Pythium*, and *Rhizoctonia*, which often occur as single or mixed infections [3]. In recent years, changes in cropping systems and planting patterns have led to an increase in the severity of root rot [4,5,6]. While chemical seed treatments and fungicides remain effective, their overuse disrupts rhizosphere microbial communities, pollutes the environment, and fosters resistance [7]. Thus, biocontrol agents, including beneficial bacteria and fungi, represent a sustainable alternative by suppressing pathogens and promoting plant health [8]. Microbial inoculants such as *Paenibacillus polymyxa*, *Bacillus subtilis*, and *Pseudomonas fluorescens* have demonstrated broad-spectrum antagonism against soil-borne pathogens and, in some cases, beneficial effects on plant growth [9,10]. However, the exploration of novel antagonistic bacteria with strong biocontrol activity and additional ecological functions remains limited.

In response to the increasing challenges posed by the evolution of fungicide resistance in pathogens, the symbiotic bacteria of entomopathogenic nematodes (EPNs) have emerged as a novel biological resource with significant development potential and promising applications [11]. Notably, these EPN-symbiotic bacteria exhibit strong antifungal activity against plant pathogens while being non-toxic to non-target organisms [12]. EPNs have been utilized for years in the biological control of agricultural pests. Extensive research indicates that the biocontrol efficacy of EPNs is primarily attributed to their bacterial symbionts [13,14]. These bacteria not only aid the nematodes in killing the insect host through the production of insecticidal proteins, immunosuppressants, and lysozymes, but also generate a diverse array of other metabolites, including pigments, fluorescent compounds, and antimicrobial agents [15,16]. Numerous studies have shown that these antimicrobial agents exhibit broad-spectrum inhibitory activity against bacteria, fungi, and yeasts. For example, metabolites produced by *X. nematophila* have been demonstrated to inhibit several plant pathogens, including *R. solani*, *Botrytis cinerea*, and *P. capsici* [17,18].

*Xenorhabdus* (Enterobacteriaceae) is a genus of Gram-negative bacteria that are obligately symbiotic with *Steinernema nematodes* [19]. *X. budapestensis*, a relatively newly described species in the genus *Xenorhabdus*, has garnered growing attention owing to its diverse metabolic potential [20]. While previous studies have documented the strong antifungal activity of *X. budapestensis* strains against several plant pathogens, no research has specifically targeted *F. oxysporum*, a primary causal agent of soybean root rot, using *X. budapestensis* as a biocontrol agent. Therefore, this study focused on the isolation and characterization of EPN symbiotic bacteria antagonistic to *F. oxysporum* in *G. max*, and on elucidating their antagonistic mechanisms, with the aim of expanding the biological control options for the management of soybean root rot.

## 2. Materials and Methods

### 2.1. Isolation and Identification of EPN Symbiotic Bacteria

Ten entomopathogenic nematode (EPN) species stored in the collection of the Plant Protection Institute (Beijing, China) were used in this study (Table S1). To isolate symbiotic bacteria, hemolymph was extracted from *Galleria mellonella* larvae that had been reared on beeswax and infected with the respective nematode strains for 16–24 h. The hemolymph was streaked onto NBTA agar plates (5 g/L sodium chloride, 0.04 g/L 2,3,5-triphenyltetrazolium chloride, 0.025 g/L bromocresol blue (Sigma-Aldrich, St. Louis, MO, USA), 3 g/L beef extract, 10 g/L peptone (Oxoid, Basingstoke, UK), and 15 g/L agar (Solarbio, Beijing, China); pH 7.2 ± 0.2) and incubated at 28 °C in the dark [11]. A single colony from NBTA medium was subcultured to obtain a pure culture of *Xenorhabdus* or *Photorhabdus*. Purity was confirmed based on colony morphology and color on fresh NBTA plates. For long-term storage, pure bacterial cultures were maintained in Luria–Bertani (LB) broth supplemented with 20% glycerol and stored at −80 °C.

Primary blue-green single colonies on NBTA plates were picked and inoculated into 20 mL of LB broth, cultured at 28 °C with shaking at 180 rpm for 24 h. Bacterial genomic DNA was extracted using a commercial DNA extraction kit (Beijing Suolaibao Technology Co., Ltd., Beijing, China). The 16S rRNA gene was amplified by PCR using universal primers (Table S2). The 50 µL PCR mixture was subjected to the following conditions: initial denaturation at 95 °C for 3 min; 30 cycles of 95 °C for 10 s, 55 °C for 15 s, and 72 °C for 60 s; and a final extension at 72 °C for 5 min. PCR products were purified and sequenced by Beijing Liuhe BGI Technology Co., Ltd. (Beijing, China). Sequencing data were analyzed using the BLAST tool (https://ftp.ncbi.nlm.nih.gov/blast/, accessed on 25 October 2022.) from the National Center for Biotechnology Information (NCBI) for initial strain identification.

### 2.2. Phenotypic Characterization of Strain XH-4

Phenotypic characterization of strain XH-4 was performed using standard microbiological methods. All reagents for physiological and biochemical tests were purchased from Coolaber Biotechnology Co., Ltd. (Beijing, China). Gram staining was conducted to determine cell wall type. Carbohydrate utilization, biochemical assays (including methyl red, Voges–Proskauer, indole production, urease activity), and enzymatic activities (amylase, cellulase, chitinase, gelatin liquefaction, casein hydrolysis, catalase) were assessed. Siderophore production and inorganic phosphate solubilization were also tested. Cell morphology was examined by scanning electron microscopy.

### 2.3. Preparation of XH-4 Fermentation Broth

A single colony of strain XH-4 was inoculated into LB broth and cultured at 28 °C with shaking at 180 rpm for 24 h to obtain a seed culture. The seed culture was then transferred to fresh LB broth at a 1% (*v*/*v*) inoculum size and incubated under the same conditions for 72 h to complete fermentation.

### 2.4. In Vitro Screening for Antagonistic Activity in Poisoned Plate Assay

The 10 bacterial strains isolated as described in Section 2.1 were screened for antagonistic activity against *F. oxysporum* using a poisoned plate assay. Each strain was cultured in LB broth at 28 °C with shaking at 180 rpm for 72 h. The cultures were then centrifuged at 10,000 rpm for 20 min at 4 °C, and the supernatants were filter-sterilized through a 0.22 µm membrane (Millipore, Burlington, MA, USA) to obtain cell-free culture supernatants. For the antagonism assay, each cell-free culture supernatant was mixed with potato dextrose agar (PDA) (Coolaber Biotechnology Co., Ltd., Beijing, China) at a concentration of 100 mL/L and poured into Petri dishes (15 mL per plate). Plates containing sterile LB broth served as controls. An 8 mm diameter mycelial plug from a 6-day-old *F. oxysporum* culture was placed at the center of each plate and incubated at 25 °C in the dark for 7 d. Colony diameter was measured using the cross method, and the percentage inhibition of mycelial growth (PIRG) was calculated according to the formula: PIRG = [(control diameter − treatment diameter)/control diameter] × 100. Each treatment was performed in triplicate. The strain exhibiting the strongest antifungal activity was selected for further studies.

### 2.5. Antagonistic Effect of XH-4 on F. oxysporum

#### 2.5.1. Preparation of Different Bacterial Formulations for Bioassays

Fermentation broth of strain XH-4 was prepared as described in Section 2.3. To obtain the bacterial cell suspension, the culture was centrifuged, and the cell pellet was resuspended in an equal volume of sterile water. Cell-free culture supernatant was prepared by filter-sterilization as described in Section 2.4.

#### 2.5.2. In Vitro Tests of XH-4 for Inhibiting Mycelial Growth of *F. oxysporum*

The inhibitory effects of the three XH-4 preparations (fermentation broth, cell-free culture supernatant, and bacterial cell suspension) on mycelial growth were evaluated using a poisoned plate assay. Each preparation was added to molten PDA (approximately 50 °C) to achieve final concentrations of 1.25, 2.5, 5, 10, 20, and 40% (*v*/*v*). Sterile LB broth and sterile distilled water were used as the corresponding controls. An 8 mm diameter mycelial plug from a 6-day-old *F. oxysporum* culture was placed at the center of each plate and incubated at 28 °C for 7 d. Colony diameters were measured, and inhibition rates were calculated as described in Section 2.4. Each treatment was performed in triplicate.

#### 2.5.3. In Vitro Tests of XH-4 for Inhibiting Spore Germination of *F. oxysporum*

The inhibitory effects of different XH-4 preparations on spore germination of *F. oxysporum* were assessed using a 96-well microtiter plate assay. Each preparation was serially diluted with sterile water to prepare working solutions at twice the desired final concentrations (i.e., 2.5, 5, 10, 20, 40, and 80%, *v*/*v*). In each well, 100 µL of the working solution was mixed with 50 µL of a *F. oxysporum* spore suspension (1 × 10^5^ spores/mL) and 50 µL of sterile sucrose solution (5 g/L), resulting in a total volume of 200 µL per well and achieving final concentrations of 1.25, 2.5, 5, 10, 20, and 40% (*v*/*v*). A relatively low spore concentration (1 × 10^5^ spores/mL) was used to facilitate accurate microscopic observation and counting of individual spore germination events. Wells containing LB broth and distilled water instead of the working solution served as controls. The microtiter plates were incubated at 28 °C for 12 h, and spore germination was observed under a light microscope. Each treatment was performed in triplicate. For each replicate, at least 200 spores were counted from three randomly selected microscopic fields. Spore germination was defined as the length of the germ tube exceeding the longitudinal length of the spore. The germination rate was calculated as the percentage of germinated spores relative to the total number of spores. The inhibition rate (%) was calculated as: [(germination rate in control − germination rate in treatment)/germination rate in control] × 100.

### 2.6. Antifungal Spectrum of Strain XH-4

The antifungal spectrum of strain XH-4 was determined using the method described in Section 2.4, with mycelial plugs from 16 different plant pathogens replacing *F. oxysporum*. The tested pathogens included *Alternaria longipes*, *F. oxysporum* f. sp. *vasinfectum*, *P. nicotianae*, *Botryosphaeria dothidea*, *Cytospora chrysosperma*, *Magnaporthe oryzae*, *Exserohilum turcicum*, *Stagonosporopsis cucurbitacearum*, *Alternaria alternata*, *Diaporthe sojae*, *Colletotrichum asianum*, *R. solani*, *F. tricinctum*, *F. asiaticum*, *Macrophomina phaseolina*, and *F. solani*. All pathogens were obtained from the culture collection of the Plant Protection Institute (Beijing, China). Each treatment was performed in triplicate.

### 2.7. Biocontrol Efficacy of XH-4 Against Soybean Root Rot Under Greenhouse Conditions

A pot experiment was conducted to evaluate the biocontrol efficacy of strain XH-4 against soybean root rot caused by *F. oxysporum* (FOX). *G. max* seeds (Zhonghuang 13, Shandong Xuhong Seed Industry Co., Ltd., Jinan, Shandong, China) were surface-sterilized with 70% ethanol for 1 min, followed by 1% (*v*/*v*) sodium hypochlorite for 2–3 min, rinsed five times with sterile distilled water, and germinated on moist sterile filter paper for 3 d. Uniform seedlings were transplanted into plastic pots (18 cm diameter) containing sterilized field soil, with five plants per pot. Plants were maintained in a greenhouse at 25 °C with a 14/12 h light/dark photoperiod. At the fully expanded second trifoliate leaf stage, seedlings were treated by root irrigation with 10 mL of XH-4 fermentation broth at concentrations of 5% or 40% (*v*/*v*). Sterile water served as the negative control, and 500 µg·mL^−1^ metalaxyl–hymexazol (30% aqueous solution, Henan Ledaofu Plant Protection Co., Ltd., Shangqiu, Henan, China) served as the positive control. After 24 h, each plant was inoculated with 10 mL of a *F. oxysporum* spore suspension (1 × 10^6^ spores/mL) applied to the root zone by soil drenching. A relatively higher spore concentration (1 × 10^6^ spores/mL) was used to ensure sufficient and uniform infection pressure for stable disease development under greenhouse conditions. Disease severity was evaluated 15 d post-inoculation using a 0–7 scale [21]: 0 = no symptoms; 1 = slight darkening of fibrous roots, aboveground growth normal; 3 = slight darkening of taproot, aboveground growth normal; 5 = severe darkening of taproot or hypocotyl erosion, poor aboveground growth; 7 = root necrosis, plant death. The disease index (DI) was calculated as: DI = [Σ (number of diseased plants at each level × rating)/(total number of plants × highest rating)] × 100. Control efficacy (%) was calculated as: [(DI_control_ − DI_treatment_)/DI_control_] × 100. Each treatment was replicated three times.

### 2.8. Assessment of Defense-Related Enzyme Activities in Soybean

To investigate whether strain XH-4 induces defense responses in soybean against *F. oxysporum*, the activities of key antioxidant and phenylpropanoid pathway enzymes were measured at 0, 24, 48, 72, and 96 h post-inoculation. *G. max* seedlings were grown and treated as described in Section 2.7. Four treatment groups were included: (1) sterile water control (Water); (2) pathogen control (Water + FOX); (3) 40% XH-4 fermentation broth alone (40% XH-4); and (4) 40% XH-4 + FOX. Leaf samples (0.1 g) were collected at each time point, immediately frozen in liquid nitrogen, and stored at −80 °C for enzyme activity assays.

For enzyme extraction, frozen leaf samples were ground in liquid nitrogen and homogenized with the extraction buffer provided in each assay kit according to the manufacturer’s instructions. The homogenate was centrifuged at 8000× *g* for 10 min at 4°C, and the supernatant was used for enzyme activity determinations. The activities of superoxide dismutase (SOD), peroxidase (POD), catalase (CAT), polyphenol oxidase (PPO), and phenylalanine ammonia-lyase (PAL) were assayed using commercial kits (Beijing Suolaibao Technology Co., Ltd., Beijing, China) following the manufacturer’s protocols. All enzyme activities were expressed as U·g^−1^ FW protein. Each treatment was performed in triplicate at each time point.

### 2.9. Genome Sequencing, Phylogenetic Analysis, and Secondary Metabolite Gene Cluster Analysis

The XH-4 genome was sequenced using a Pacbio sequel II and DNBSEQ platform at the Beijing Genomics Institute (BGI, Shenzhen, China). Four SMRT cells Zero-Mode Waveguide arrays of sequencing were used by the PacBio platform to generate the subreads set. PacBio subreads (length < 1 kb) were removed. The program Canu was used for self-correction. Draft genomic unitigs, which are uncontested groups of fragments, were assembled using the Canua high-quality corrected circular consensus sequence subreads set. To improve the accuracy of the genome sequences, GATK (https://www.broadinstitute.org/gatk/, accessed on 6 November 2023) was used to make single-base corrections. Genome annotation was performed by BLAST alignment against multiple databases, including KEGG (Kyoto Encyclopedia of Genes and Genomes), COG (Clusters of Orthologous Groups), NR (Non-Redundant Protein Database), and GO (Gene Ontology). For phylogenetic analysis, genome assemblies of 11 closely related Xenorhabdus strains were downloaded from NCBI. Pairwise average nucleotide identity (ANI) values were calculated using FastANI with default parameters. The resulting ANI matrix was visualized as a heatmap using TBtools (version 1.120, South China Agricultural University, Guangzhou, China). ANI values were interpreted according to the established species boundary (approximately 95–96% ANI) to confirm the taxonomic affiliation of strain XH-4. The biosynthetic potential for secondary metabolite production was analyzed using AntiSMASH version 8.0 (https://antismash.secondarymetabolites.org, accessed on 28 November 2023) [22].

## 3. Results

### 3.1. Identification and Phenotypic Characterization of Bacterial Antagonists

**Figure 1:**
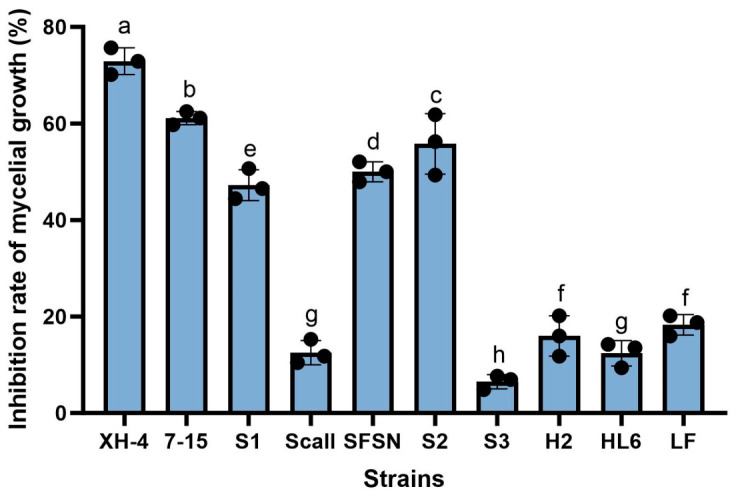
Antifungal effect of cell-free culture supernatants from ten EPN symbiotic bacteria against *Fusarium oxysporum*. Bars represent the mean inhibition rate (%) of mycelial growth for each strain, and error bars indicate the standard deviation (SD). Black circles represent individual biological replicates (*n* = 3). Bars sharing different letters indicate statistically significant differences (*p* < 0.05). Statistical significance among treatments was determined by one-way ANOVA followed by Tukey’s multiple comparison test.

A total of 10 symbiotic bacterial strains were isolated from different entomopathogenic nematodes. Based on 16S rRNA gene sequence analysis, these isolates were identified as members of the genera *Xenorhabdus* and *Photorhabdus*, showing sequence similarities ranging from 92.46% to 99.53% with their closest type strains (Table S1). Poisoned plate assays demonstrated that all tested strains exhibited varying degrees of inhibitory activity against the mycelial growth of *F. oxysporum* (Figure 1). Among them, strain XH-4 showed the strongest antifungal activity, with a mycelial growth inhibition rate of 72.92%. Strains 7–15, S2, and SFSN also displayed relatively high inhibitory effects, with inhibition rates above 50%. In contrast, strains S3, Scall, H2, HL6, and LF exhibited comparatively weak antagonistic activity, with inhibition rates generally below 20%. Based on these results, strain XH-4 was selected for further phenotypic, molecular, and biocontrol analyses.

The single colony that grew on LB agar plate was smooth, convex, and cream-colored after 3 d at 28 °C; the primary phase colonies of XH-4 were dark blue on NBTA indicator plates (Figure S1a,b). Gram staining revealed that XH-4 was a Gram-negative, rod-shaped bacterium (Figure S1c), which was further confirmed by scanning electron microscopy, showing short rod-shaped cells (Figure S1d). Biochemical assays showed that XH-4 was positive for maltose and D-glucose utilization, gelatin liquefaction, cellulase, chitinase, siderophore production, and inorganic phosphate solubilization, but negative for sucrose utilization, methyl red, Voges–Proskauer, casein hydrolysis, amylase, catalase, indole production, and urease activity (Table S3; Figure S2).

### 3.2. Inhibitory Effects of Different XH-4 Preparations on Mycelial Growth of F. oxysporum

The effects of the fermentation broth, cell-free culture supernatant, and bacterial cell suspension on the mycelial growth of *F. oxysporum* were evaluated using a poisoned plate assay (Table 1). Both the fermentation broth and cell-free culture supernatant inhibited mycelial growth in a concentration-dependent manner. For the fermentation broth, the inhibition rate increased from 26.62% at 1.25% (*v*/*v*) to 88.19% at 40% (*v*/*v*), exceeding 70% at concentrations ≥10%. Similarly, the cell-free culture supernatant exhibited inhibition ranging from 32.18% to 83.80% across the same concentration range, with no significant difference observed between the two treatments at higher concentrations (≥20%). In contrast, the bacterial cell suspension showed weak inhibitory effects, with inhibition rates remaining below 20% at concentrations ≤10% and reaching only 57.64% at the highest concentration tested (40%).

### 3.3. Inhibitory Effects of Different XH-4 Preparations on Spore Germination of F. oxysporum

The inhibitory effects of the different XH-4 preparations on spore germination were summarized in Table 1. Spore germination was highly sensitive to the fermentation broth and cell-free culture supernatant. The fermentation broth inhibited germination by 88.81% at the lowest concentration tested (1.25%, *v*/*v*) and achieved complete (100%) inhibition at concentrations ≥20%. The cell-free culture supernatant also completely suppressed germination at concentrations ≥10%. Conversely, the bacterial cell suspension had a limited effect on spore germination. No significant inhibition was observed at concentrations ≤10%, and only partial inhibition (38.96%) was detected at the maximum concentration of 40%.

### 3.4. Morphological Alterations of F. oxysporum Hyphae Induced by XH-4 Treatment

**Figure 2:**
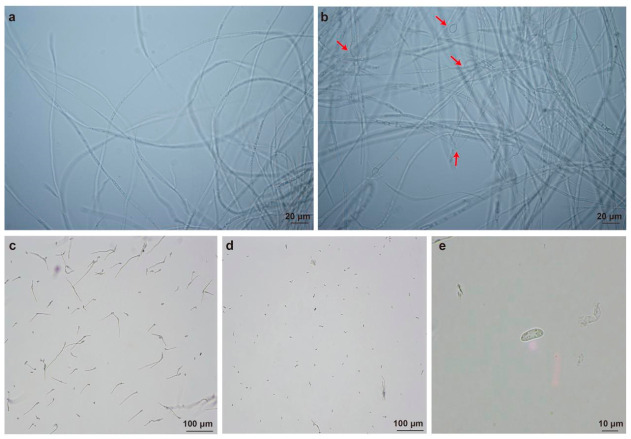
Microscopic observation of *Fusarium oxysporum* treated with cell-free culture supernatant. (**a**) Hyphae in the control group. (**b**) Hyphae after treatment with 10% (*v*/*v*) cell-free culture supernatant; red arrows indicate hyphal swelling and narrowing. (**c**) Spore germination in the control group. (**d**) Spores in the treatment group. (**e**) Enlarged view of a morphologically altered spore from the treatment group. Scale bars: 20 μm (**a**,**b**); 100 μm (**c**,**d**); 10 μm (**e**).

Microscopic examination revealed distinct morphological differences between the control and treatment groups (Figure 2). In the control group, hyphae were smooth, slender, and uniformly distributed, with a consistent diameter and intact structure, showing no obvious swelling or abnormal thickening (Figure 2a). In contrast, hyphae in the treatment group exhibited visible structural alterations, including localized swelling and irregular thickening along the filaments (red arrows, Figure 2b), resulting in uneven diameters compared to the control. Regarding spore germination, fungal spores in the control group exhibited normal development, with elongated germ tubes and typical morphology (Figure 2c). In the treatment group, fewer germinated spores were observed within the same field of view (Figure 2d). An enlarged view of a representative treated spore (Figure 2e) revealed an irregular shape and uneven intracellular distribution, with internal contents appearing condensed and granular.

### 3.5. Broad-Spectrum Antifungal Activity of XH-4

**Figure 3:**
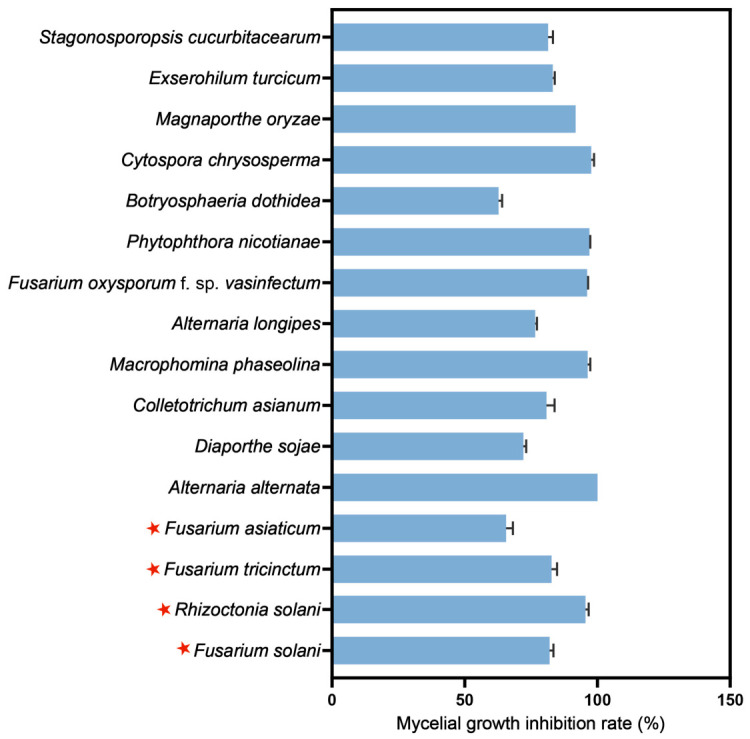
Broad-spectrum antifungal activity of XH-4 cell-free culture supernatant against plant pathogenic fungi. Red stars represent pathogenic fungi causing soybean root rot.

The cell-free culture supernatant of strain XH-4 exhibited the broad-spectrum antifungal activity against all 16 phytopathogenic fungi tested (Figure 3; Figure S3). Notably, it strongly inhibited several key root rot pathogens (red asterisks, Figure 3), including *F. solani*, *R. solani*, *F. tricinctum*, and *F. asiaticum*, with mycelial growth inhibition rates generally exceeding 50%. The strongest inhibition (100%) was observed against *A. alternata*, while the weakest, though still substantial, was against *B. dothidea* (62.76%). These results highlighted the significant potential of XH-4 in managing a broad spectrum of plant diseases.

### 3.6. Biocontrol Effect of XH-4 Against Soybean Root Rot Under Greenhouse Conditions

**Figure 4:**
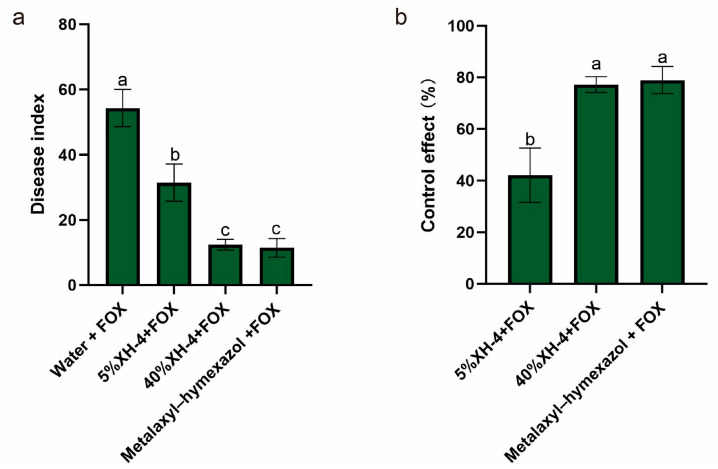
Statistical analysis of disease suppression by XH-4 fermentation broth. (**a**) Disease index. (**b**) Control efficacy. Soybean seedlings were treated with sterile water, XH-4 at different concentrations (5% and 40%, *v*/*v*), or the chemical fungicide metalaxyl–hymexazol, followed by inoculation with *Fusarium oxysporum* (FOX). Values represent mean ± SD. Different lowercase letters indicate significant differences (*p* < 0.05, Tukey’s test).

**Figure 5:**
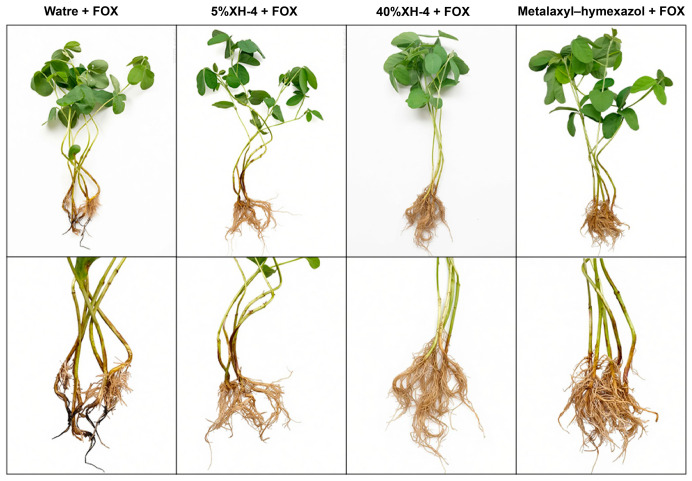
Representative disease symptoms and root morphology under different treatments.

The biocontrol efficacy of XH-4 against *F. oxysporum* (FOX) was evaluated under greenhouse conditions (Figure 4). The pathogen control (Water + FOX) developed severe disease, with a disease index of 54.28 (Figure 4a). Treatment with XH-4 reduced disease severity in a concentration-dependent manner. The 5% XH-4 treatment decreased the disease index to 31.43, while the 40% XH-4 treatment decreased it to 12.38, a value comparable to that of the metalaxyl–hymexazol treatment (11.43). Statistical analysis confirmed that the 40% XH-4 and metalaxyl–hymexazol treatments did not differ significantly, and both were more effective than the 5% XH-4 treatment (*p* < 0.05). Consistent with these results, control efficacy increased with XH-4 concentration (Figure 4b). The 5% and 40% XH-4 treatments achieved control efficacies of 42.11% and 77.19%, respectively, with the latter showing no significant difference from metalaxyl–hymexazol (78.95%). Representative images (Figure 5) corroborated these findings. Plants treated with 40% XH-4 or metalaxyl–hymexazol exhibited healthier root systems and improved growth compared to the pathogen control, which showed severe root browning and stunted development.

### 3.7. Induction of Soybean Defense Responses by XH-4 Under Pathogen Challenge

**Figure 6:**
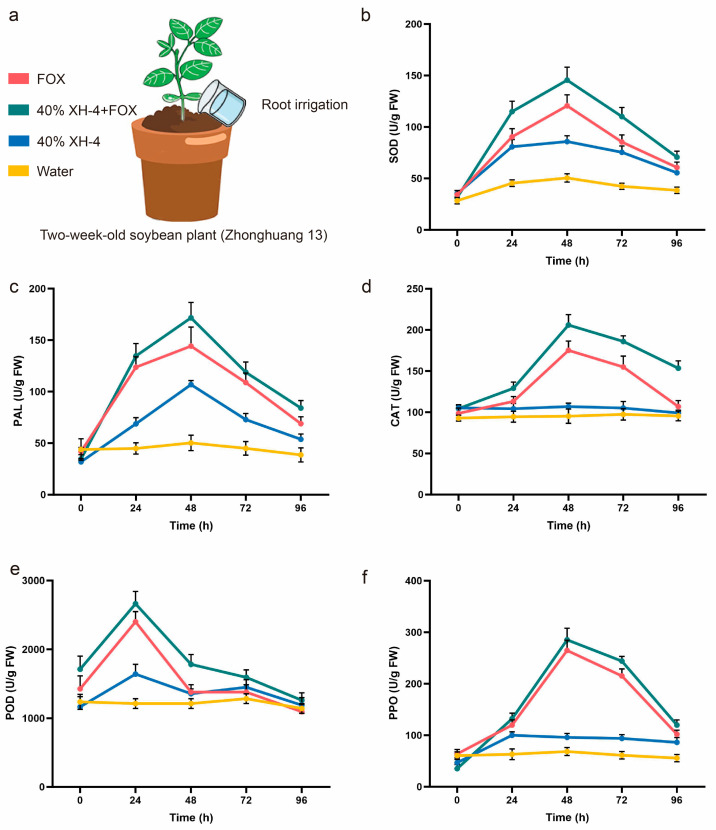
Effects of XH-4 fermentation broth treatment on defense-related enzyme activities in soybean under *Fusarium oxysporum* infection. (**a**) Experimental design showing root irrigation treatments applied to two-week-old soybean plants (cv. Zhonghuang 13). (**b**) Superoxide dismutase (SOD) activity. (**c**) Phenylalanine ammonia-lyase (PAL) activity. (**d**) Catalase (CAT) activity. (**e**) Peroxidase (POD) activity. (**f**) Polyphenol oxidase (PPO) activity.

To determine if strain XH-4 enhanced host defense responses during *F. oxysporum* infection, we monitored the activities of key antioxidant and phenylpropanoid enzymes (Figure 6). Following pathogen inoculation, the combined 40% XH-4 + FOX treatment consistently induced higher enzyme activities than FOX treatment alone across all time points. Specifically, SOD activity peaked at 48 h, with the combined treatment showing enhanced ROS scavenging capacity compared to the pathogen-only control (Figure 6b). Similarly, PAL, CAT, POD, and PPO activities were more strongly activated during the early infection phase (24–48 h) in the combined treatment group (Figure 6c–f). Although activities gradually declined thereafter, they remained elevated relative to the untreated control.

### 3.8. Assembly and Annotation of the XH-4 Genome Sequence

**Figure 7:**
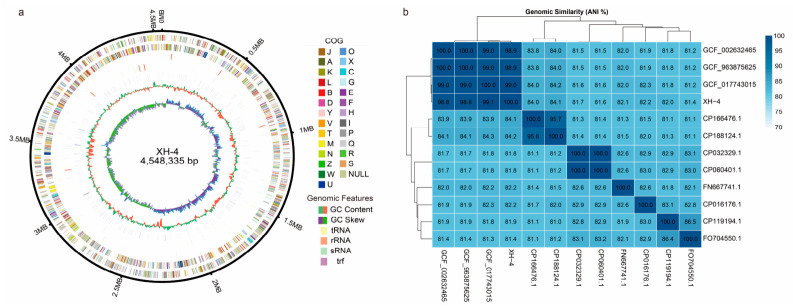
Genome features and average nucleotide identity (ANI) analysis of strain XH-4. (**a**) Circular representation of the XH-4 genome. From outer to inner rings: genome size; forward- and reverse-strand coding sequences colored by COG categories; forward-and reverse-strand ncRNAs; repeat regions; GC content; and GC skew. (**b**) Average nucleotide identity (ANI) heatmap showing genomic similarity between strain XH-4 and related reference genomes. Color intensity represents ANI values (%). CP166476.1 (*Xenorhabdus stockiae* strain RT25.5); CP188124.1 (*Xenorhabdus stockiae* strain HN_xs01); FO704550.1 (*Xenorhabdus doucetiae* str. FRM16); CP016176.1 (*Xenorhabdus hominickii* strain ANU1); CP119194.1 (*Xenorhabdus bakwenae* strain SF857) CP032329.1 (*Xenorhabdus nematophila* strain YL001) CP060401.1 (*Xenorhabdus nematophila* strain SII); FN667741.1 (*Xenorhabdus bovienii* SS-2004); GCA_002632465.1 (*Xenorhabdus budapestensis* strain DSM 16342); GCA_017743015.1 (*Xenorhabdus budapestensis* C-7-2); GCA_963875625.1 (*Xenorhabdus budapestensis* ena-yuan-GCF_002632465.1).

To elucidate the taxonomic status and genetic basis of antagonism in strain XH-4, its complete genome was sequenced using a combination of DNBSEQ short-read and Oxford Nanopore long-read technologies. This process yielded 1310 Mb of raw data, from which 1238 Mb of high-quality data were assembled into a single, complete circular chromosome of 4,548,335 bp with a GC content of 43.20% (Figure 7a). Genome annotation predicted 4315 protein-coding genes, 81 tRNAs, 22 rRNAs, and 33 sRNAs, along with one CRISPR system (Table S4). Average nucleotide identity (ANI) analysis confirmed the species-level identification of XH-4 as *X. budapestensis*, showing high ANI values (98–99%) with reference strains of the same species, significantly above the species cutoff (95–96%), and lower values (81–84%) with more distantly related species (Figure 7b).

### 3.9. The Prediction of Secondary Metabolites in Xenorhabdus budapestensis XH-4

**Figure 8:**
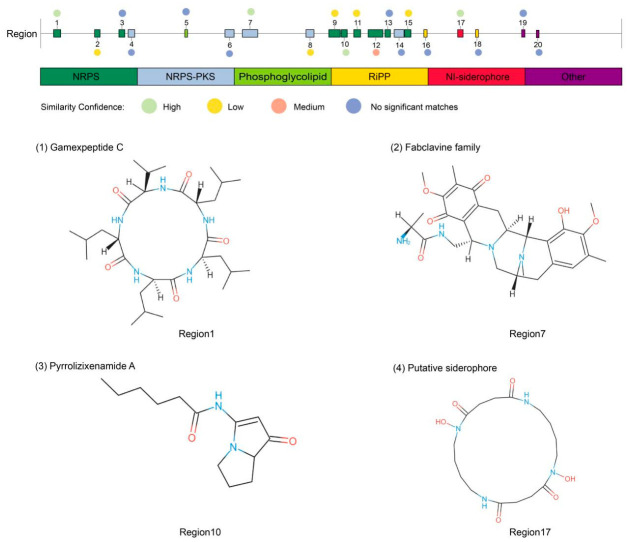
Predicted biosynthetic gene clusters (BGCs) for secondary metabolites in the genome of *Xenorhabdus budapestensis* XH-4. Different colors indicate distinct classes of BGCs, including nonribosomal peptide synthetases (NRPS), NRPS–polyketide synthase (NRPS–PKS) hybrids, phosphoglycolipid, ribosomally synthesized and post-translationally modified peptides (RiPPs), NRPS-independent siderophores, and other types. Circles above the clusters indicate the similarity confidence to known clusters (high, medium, low, or no significant matches). Representative chemical structures of selected predicted products are shown, including (1) Gamexpeptide C (Region 1), (2) Fabclavine family compounds (Region 7), (3) Pyrrolizixenamide A (Region 10), and (4) Siderophore (Region 17).

AntiSMASH analysis of the complete XH-4 genome identified 20 biosynthetic gene clusters (BGCs) associated with secondary metabolite production (Figure 8; Table 2), which included clusters for nonribosomal peptide synthetases (NRPS), NRPS-polyketide synthase (PKS) hybrids, RiPPs, and siderophores. Four clusters showed high similarity (100%) to known compound biosynthetic pathways, including those for gamexpeptide C (Region 1), fabclavine (Region 7), and pyrrolizixenamide A (Region 10). Additionally, a siderophore cluster (Region 17) was identified. The presence of multiple BGCs with homology to known antimicrobials, alongside several clusters with low or no similarity to characterized pathways, provides a strong genomic basis for the observed antifungal activity of XH-4 and suggests potential for novel bioactive compound discovery.

## 4. Discussion

Soybean root rot, caused by a complex of pathogens including *Fusarium* spp. and *Phytophthora sojae*, causes substantial yield losses [23]. *F. oxysporum* is a major pathogen of this disease, and its wide distribution, high pathogenicity, and genetic diversity complicate effective management [24,25,26]. Biological control offers a sustainable alternative to chemical fungicides. Previous work showed that metabolites from *X. bovienii* suppressed *F. solani* [27]. Consistent with this, *X. budapestensis* XH-4 significantly inhibited *F. oxysporum* mycelial growth in poisoned plate assays and protected soybean plants under greenhouse conditions, with the efficacy comparable to the chemical fungicide (Figure 4; Table 1). These results indicated that *Xenorhabdus* bacteria were a promising source of bioactive metabolites for soybean root rot control.

Biocontrol agents with broad-spectrum antagonistic activity have greater practical value because they can control multiple diseases, increasing their potential for commercial application. In this study, *X. budapestensis* XH-4, a symbiotic bacterium isolated from an EPN, showed strong antifungal activity against a wide range of plant pathogens, including *F. oxysporum*, *R. solani*, *A. alternata*, etc., which belong to different taxonomic groups and have different infection modes. The commercial potential of broad-spectrum biocontrol agents is well illustrated by examples such as *P. aeruginosa* and *B. stratosphericus*, both of which have been developed to suppress multiple plant pathogens [28]. Beyond agricultural applications, cell-free culture supernatant of *X. budapestensis* has also shown activity against clinical and zoonotic pathogens [11,29,30], suggesting broader utility. While EPN symbiotic bacteria are primarily known for their insecticidal activity in association with nematodes, their potential for plant disease control and other applications remains underexplored. These findings warrant further investigation into the biocontrol mechanisms of *X. budapestensis* XH-4 and the strain potential for commercial development in sustainable agriculture.

The antifungal activity of *X. budapestensis* XH-4 appears to involve multiple mechanisms. First, cell-free culture supernatant causes severe morphological damage to *F. oxysporum* hyphae and strongly inhibited spore germination, indicating that secreted metabolites play a key role. These observations are consistent with studies showing that *Xenorhabdus* spp. produce extracellular compounds that disrupt fungal cell wall integrity and membrane function [31,32]. Second, *X. budapestensis* XH-4 is tested positive for cellulase and chitinase activities. Chitinases hydrolyze chitin, a major component of fungal cell walls, while cellulases may act on other cell wall polysaccharides [33,34]. Similar enzymatic activities have been reported in other *Xenorhabdus* strains. For instance, treatment with sterile supernatant from *X. budapestensis* C72 induced plasmolysis in *Bipolaris cinerea* spores and partial mycelial death, while the bacterium produced chitinase, cellulase, and β-1,3-glucanase [11,31]. These observations suggest that the direct inhibition of *F. oxysporum* by *X. budapestensis* XH-4 may involve both diffusible metabolites and hydrolytic enzymes.

Beyond direct pathogen inhibition, *X. budapestensis* XH-4 also enhanced plant defense responses. Under pathogen challenge, treatment with XH-4 significantly increased the activities of antioxidant enzymes (SOD, CAT, POD) and phenylpropanoid pathway enzymes (PAL, PPO) compared to the pathogen-only control. Elevated antioxidant enzyme activity helps reduce reactive oxygen species (ROS) accumulation, thereby limiting oxidative damage during infection [35,36]. PAL is a key enzyme in the phenylpropanoid pathway, which produces antimicrobial compounds and signaling molecules involved in systemic resistance [37]. Similar defense induction has been reported with other biocontrol strains, such as *Bacillus* sp. IPR-4 and *Carnobacterium* sp. SJ-5 [37,38]. These results indicate that *X. budapestensis* XH-4 not only suppresses the pathogen directly but also primes the plant’s defense system, contributing to the strain overall biocontrol efficacy.

The antagonistic activity of *X. budapestensis* XH-4 involves multiple mechanisms, including antimicrobial metabolites, nutrient competition, and induction of plant defense. Bacteria of the genus *Xenorhabdus* are recognized as important sources of bioactive natural products, with genomes enriched in NRPS, PKS, and hybrid biosynthetic systems that enable synthesis of structurally diverse antimicrobial compounds [39,40]. In this study, four clusters showed relatively high similarity to known BGCs, and three of these were associated with antagonistic activity against bacterial and fungal pathogens, including fabclavine, pyrrolizixenamide A, and putrebactin/avaroferrin. Putrebactin/avaroferrin are siderophores produced by XH-4 that chelate ferric iron (Fe^3+^) under iron-limiting conditions, thereby enhancing plant growth and disease resistance [41,42]. Fabclavines are peptide–polyketide–polyamino hybrid compounds produced by pathogenic bacteria that exhibit broad-spectrum activity against fungi, bacteria, and protozoa [43]; previous studies have demonstrated their significant inhibitory effects against the wheat pathogen *F. graminearum* [44]. Pyrrolizixenamide A is a nonribosomal peptide synthetase (NRPS)-derived lipopeptide alkaloid with reported antibacterial and antitumor activities [45]. In addition, several other predicted BGCs showed similarity to known antimicrobial clusters. For example, xenocoumacin have been reported to inhibit plant pathogens such as *B. cinerea* and *P. infestans* [43], while nematophin exhibits inhibitory activity against *R. solani* and *P. infestans* [43]. Notably, multiple predicted BGCs displayed low or no similarity to previously characterized clusters, suggesting that XH-4 might possess the potential to produce novel antimicrobial secondary metabolites. The diverse BGCs identified in the XH-4 genome thus provide a genetic basis for its broad-spectrum antifungal activity, consistent with the recognized potential of *Xenorhabdus* spp. as rich sources of new bioactive secondary metabolites [40].

The following limitations should be considered when interpreting these results. The biocontrol efficacy of microbial agents under field conditions is often inconsistent due to environmental factors such as temperature, humidity, and soil microbiota [46]. EPN-symbiotic bacteria are not adapted for long-term survival in soil without their nematode hosts, which may limit their persistence and efficacy in agricultural settings. Future studies should evaluate the performance of *X. budapestensis* XH-4 under field conditions, assess the strain compatibility with other agricultural practices, and investigate formulations that can enhance its stability and delivery. Additionally, further characterization of the unidentified BGCs may lead to the discovery of novel antifungal compounds.

## 5. Conclusions

In conclusion, *X. budapestensis* XH-4 shows strong biocontrol potential against *F. oxysporum* through a dual mode of action involving direct antifungal activity and induction of plant defense responses. The strain broad-spectrum activity and rich biosynthetic gene cluster repertoire make it a promising candidate for further development as a biocontrol agent against soybean root rot and other fungal diseases.

## Figures and Tables

**Table 1:** Inhibitory effects of fermentation broth, cell-free culture supernatant, and bacterial cell suspension at different concentrations on mycelial growth and spore germination of *Fusarium oxysporum*.

Treatment	Concentration % (*v*/*v*)	Inhibition Rate of Mycelial Growth (%)	Inhibition Rate of Spore Germination (%)
Fermentation broth	1.25	26.62 ± 0.87 h	88.81 ± 2.79 b
	2.5	28.94 ± 0.33 gh	93.35 ± 1.31 ab
	5	37.96 ± 0.87 f	92.83 ± 1.08 ab
	10	72.45 ± 1.18 c	96.50 ± 0.89 ab
	20	83.10 ± 0.65 ab	100.00 ± 0.00 a
	40	88.19 ± 3.00 a	100.00 ± 0.00 a
Cell-free culture supernatant	1.25	32.18 ± 1.43 g	92.83 ± 0.65 ab
	2.5	42.36 ± 0.57 f	94.05 ± 1.31 ab
	5	48.61 ± 2.60 e	94.40 ± 0.25 ab
	10	71.76 ± 1.64 c	100.00 ± 0.00 a
	20	80.56 ± 2.84 b	100.00 ± 0.00 a
	40	83.80 ± 1.18 ab	100.00 ± 0.00 a
Bacterial cell suspension	1.25	15.51 ± 0.33 i	0.00 ± 0.00 e
	2.5	16.67 ± 1.13 i	0.00 ± 0.00 e
	5	17.13 ± 1.18 i	1.36 ± 3.81 e
	10	30.56 ± 0.57 gh	0.00 ± 0.00 e
	20	42.82 ± 0.33 ef	23.40 ± 7.82 d
	40	57.64 ± 0.98 d	38.96 ± 2.02 c

Different lowercase letters in each column indicate statistically significant differences among treatments as determined by Tukey’s multiple range test (*p* < 0.05).

**Table 2:** Identified secondary metabolite regions in genome of strain XH-4.

Region	Type	From	To	Most Similar Known Cluster	Similarity	MIBiG Accession	Gene Cluster from Organisms
Region 1	NRPS	101,441	157,073	gamexpeptide C	100%	BGC0001128	*Photorhabdus laumondii* subsp. laumondii TTO1
Region 2	NRPS-like	421,519	464,680	safracin A/safracin B	20%	BGC0000421	*Pseudomonas fluorescens*
Region 3	NRPS	612,373	657,931	B/ashimides A/ashimide A /ashimide B	10%	BGC0002288	*Streptomyces* sp. NA03103
Region 4	NRPS, T1PKS	684,056	736,645				
Region 5	phosphoglycolipid	1,127,647	1,149,617				
Region 6	NRPS, T1PKS	1,442,483	1,513,588	xenocoumacin I/xenocoumacin II	14%	BGC0001054	*Xenorhabdus nematophila* ATCC 19061
Region 7	hglE-KS, PUFA, NRPS, T1PKS, NRPS-like	1,578,469	1,699,102	Fabclavine Ia /fabclavine Ib/fabclavine IIa/fabclavine IIb	100%	BGC0001130	*Xenorhabdus budapestensis*
Region 8	NRP-metallophore, NRPS, T1PKS	2,076,305	2,139,227	HTTPCA/prepiscibactin/piscibactin	24%	BGC0002715	*Photorhabdus laumondii* subsp. laumondii
Region 9	NRPS	2,255,143	2,345,097	stechlisin B2/stechlisin C3/stechlisin D3/tensin/stechlisin E2/stechlisin F	50%	BGC0002092	*Pseudomonas* sp.
Region 10	NRPS	2,352,206	2,399,372	pyrrolizixenamide A	100%	BGC0001873	Xenorhabdus szentirmaii DSM 16338
Region 11	NRPS	2,449,931	2,504,148	nematophin	40%	BGC0001692	*Xenorhabdus nematophila* ATCC 19061
Region 12	NRPS, NRPS-like	2,563,714	2,678,724	xeneprotides A–C	66%	BGC0001826	*Xenorhabdus* sp. KJ12.1
Region 13	NRPS	2,694,472	2,737,879	piscibactin	12%	BGC0002533	*Photobacterium damselae* subsp. piscicida DI21
Region 14	transAT-PKS, NRPS	2,766,707	2,843,462				
Region 15	NRPS	2,846,487	2,897,550	xenoamicin A /xenoamicin B	25%	BGC0000464	*Xenorhabdus doucetiae*
Region 16	thiopeptide	2,998,825	3,024,808				
Region 17	NI-siderophore	3,262,998	3,305,655	putrebactin/avaroferrin	100%	BGC0001870	*Xenorhabdus budapestensis*
Region 18	LAP	3,403,306	3,426,348				
Region 19	betalactone	3,763,720	3,789,363	lipopolysaccharide	5%	BGC0000774	*Xanthomonas campestris* pv. campestris
Region 20	terpene-precursor	3,879,694	3,900,611				

## Data Availability

Genome sequence data base of *Xenorhabdus budapestensis* XH-4 as discussed in the manuscript have been deposited in NCBI Database and are accessible through Accession Nos. ASM4808165.

## Supplementary Materials

The following supporting information can be downloaded at: https://www.mdpi.com/article/10.3390/jof12050332/s1, Figure S1: Morphological characteristics of strain XH-4. a: Colony morphology on LB agar plate; b: Colony morphology on NBTA agar plate; c: Gram staining of XH-4 cells; d: Electron micrograph showing the cell morphology of XH-4. Figure S2: Physiological and biochemical characteristics of strain XH-4. Figure S3: Broad-spectrum antifungal activity of strain XH-4 against diverse phytopathogenic fungi. Table S1: Identification of *Xenorhabdus* and *Photohabdus* strains based on 16S rRNA gene sequence analysis. Table S2: The primer sequences for the 16S rRNA. Table S3: Physiological and biochemical characteristics of strain XH-4. Table S4. General characteristics of the strain XH-4 genome.
