# Supplementary material for: Genomic Insights and Antifungal Efficacy of Xenorhabdus budapestensis XH-4 in Combating Soybean Root Rot
Source: J Fungi (Basel). 2026 May 2;12(5):332. doi: 10.3390/jof12050332 (PMC13208338; doi:10.3390/jof12050332)
Supplement: Supplementary file 1 [file jof-12-00332-s001.zip › jof-4207590-supplementary.pdf]

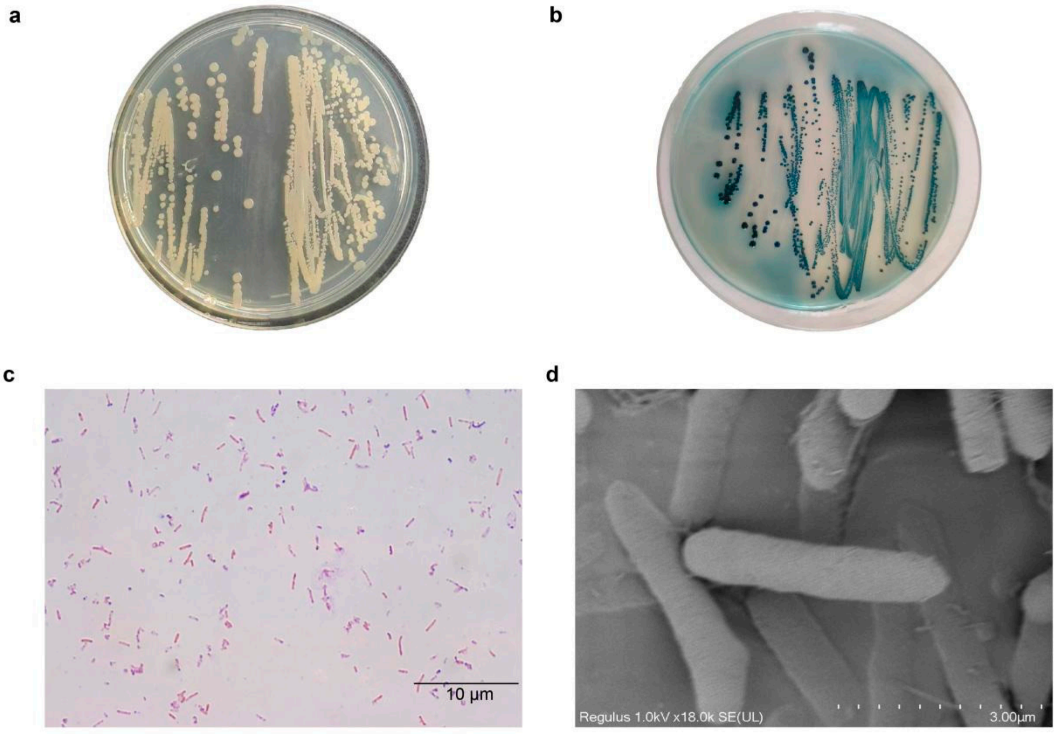

Figure S1. Morphological characteristics of strain XH-4. a: Colony morphology on LB agar plate; b: Colony morphology on NBTA agar plate; c: Gram staining of XH-4 cells; d: Electron micrograph showing the cell morphology of XH-4.

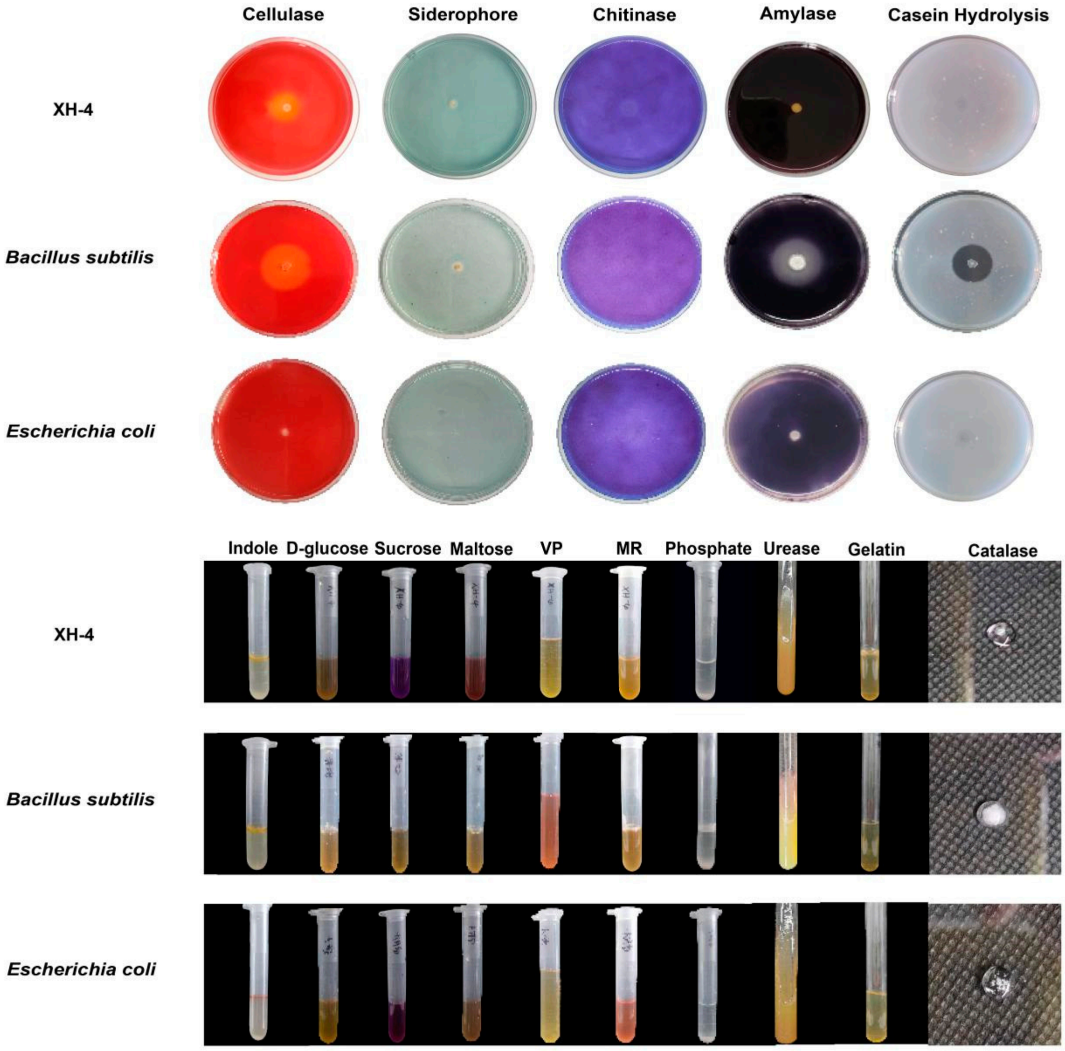

Figure S2. Physiological and biochemical characteristics of strain XH-4.

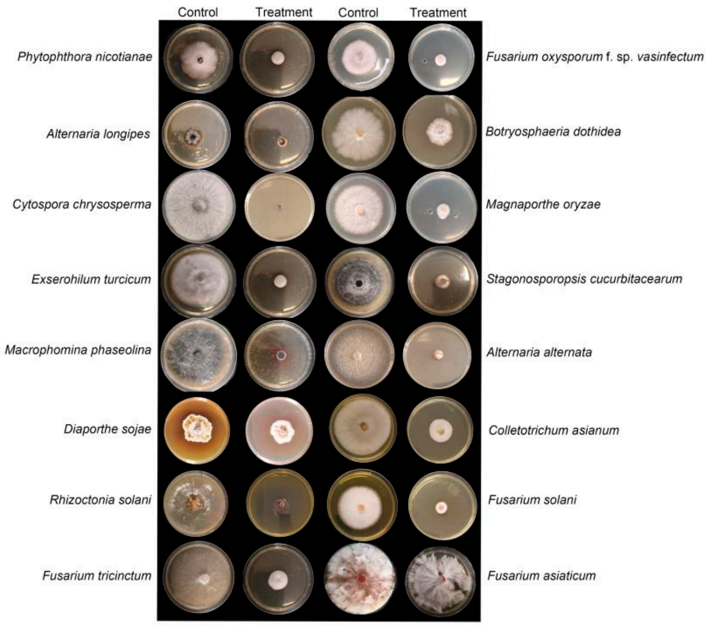

Figure S3. Broad-spectrum antifungal activity of strain XH-4 against diverse phytopathogenic fungi.

Table S1. Identification of *Xenorhabdus* and *Photohabdus* strains based on 16S rRNA gene sequence analysis.

Strains	Closest species	Sequence similarity (%)	Source: Entomopathogenic nematode	Collection site
7-15	*Xenorhabdus budapestensis* strain BIC	99.49	*Steinernema ceratophorum*	Changping, Beijing, China
XH-4	*Xenorhabdus budapestensis* strain DSM 16342	99.52	*Steinernema ceratophorum*	Rwanda, Africa
S1	*Xenorhabdus nematophila* strain A41	98.88	*Steinernema carpocapsae*	Miyun, Beijing, China
Scall	*Xenorhabdus nematophila* strain LZ-G7	92.46	*Steinernema carpocapsae*	Guyuan, Zhangjiakou, China
SFSN	*Xenorhabdus nematophila* strain UNPB73	98.21	*Steinernema carpocapsae*	Huai'an, Zhangjiakou, China
S2	*Xenorhabdus ehlersii* strain SN22	99.53	*Steinernema litorale*	Tongzi, Guizhou, China
S3	*Xenorhabdus bovienii* strain BJFS526	99.31	*Steinernema longicaudum*	Yanqing, Beijing, China
H2	*Photorhabdus kleinii* strain DSM 23513	97.66	*Heterorhabditis beicherriana*	Zhangbei, Zhangjiakou, China
HL6	*Photorhabdus kleinii* strain DSM 23513	98.29	*Heterorhabditis bacteriophora*	Huai'an, Zhangjiakou, China
LF	*Photorhabdus luminescens* subsp. *kayaii* strain ITH-LA3	98.47	*Heterorhabditis beicherriana*	Fengning Manchu Autonomou, Chengde, China

Table S2. The primer sequences for the 16S rRNA.

Primer		Sequence
16S rRNA	27F	5′-AGAGTTTGATCCTGGCTCAG-3′
	1492R	5′-TACGGYTACCTTGTTACGACTT-3′

Table S3. Physiological and biochemical characteristics of strain XH-4.

NO.	Test	XH-4	*Escherichia coli* （reference strain）	*Bacillus subtilis* （reference strain）
1	Maltose	+	+	+
2	D-glucose	+	+	+
3	Sucrose	-	-	+
4	Methyl Red (MR)	-	+	-
5	Voges-Proskauer (VP)	-	-	+
6	Casein Hydrolysis Test	-	-	+
7	Gelatin Liquefaction Test	+	-	-
8	Amylase Test	-	-	+
9	Catalase Test	-	+	+
10	Cellulase Test	+	-	+
11	Chitinase Test	+	-	+
12	Siderophore Test	+	+	+
13	Indole Test	-	+	-
14	Inorganic Phosphate Solubilization Test	+	-	+
15	Urease Test	-	-	+

Physiological and biochemical traits of strain XH-4 were compared with those of the reference strains Escherichia coli and Bacillus subtilis. “+” indicates a positive reaction, and “－” indicates a negative reaction.

Table S4. General characteristics of the strain XH-4 genome.

Chromosome	
Genome Size (bp)	4,548,335
Protein-coding genes	4,315
G + C content (%)	43.20
Copy number of tRNA	81
Copy number of rRNA	22
Copy number of sRNA	33
CRISPR Length (bp)	100
